# A case of infectious heterotopic ossification in the appendectomy scar, which formed an inflammatory granuloma

**DOI:** 10.1093/jscr/rjac370

**Published:** 2022-08-17

**Authors:** Chie Hayashi, Yusuke Takahashi, Kiyoshi Mori, Kenji Kawai, Masaaki Miyo, Reishi Toshiyama, Kenji Sakai, Takuya Hamakawa, Takashi Doi, Atsushi Takeno, Kunihito Gotoh, Michihiko Miyazaki, Koji Takami, Motohiro Hirao, Takeshi Kato

**Affiliations:** Department of Surgery, National Hospital Organization Osaka National Hospital, Chuo-ku, Osaka, Japan; Department of Surgery, National Hospital Organization Osaka National Hospital, Chuo-ku, Osaka, Japan; Department of Central Laboratory and Surgical Pathology, National Hospital Organization Osaka National Hospital, Chuo-ku, Osaka, Japan; Department of Surgery, National Hospital Organization Osaka National Hospital, Chuo-ku, Osaka, Japan; Department of Surgery, National Hospital Organization Osaka National Hospital, Chuo-ku, Osaka, Japan; Department of Surgery, National Hospital Organization Osaka National Hospital, Chuo-ku, Osaka, Japan; Department of Surgery, National Hospital Organization Osaka National Hospital, Chuo-ku, Osaka, Japan; Department of Surgery, National Hospital Organization Osaka National Hospital, Chuo-ku, Osaka, Japan; Department of Surgery, National Hospital Organization Osaka National Hospital, Chuo-ku, Osaka, Japan; Department of Surgery, National Hospital Organization Osaka National Hospital, Chuo-ku, Osaka, Japan; Department of Surgery, National Hospital Organization Osaka National Hospital, Chuo-ku, Osaka, Japan; Department of Surgery, National Hospital Organization Osaka National Hospital, Chuo-ku, Osaka, Japan; Department of Surgery, National Hospital Organization Osaka National Hospital, Chuo-ku, Osaka, Japan; Department of Surgery, National Hospital Organization Osaka National Hospital, Chuo-ku, Osaka, Japan; Department of Surgery, National Hospital Organization Osaka National Hospital, Chuo-ku, Osaka, Japan

## Abstract

Inflammatory granulomas often develop in surgical scars due to the presence of foreign bodies, such as sutures. These granulomas are called Schloffer’s tumors. Here, we report a case of heterotopic ossification(HO) in an appendectomy scar that formed an inflammatory granuloma following HO infection. A 90-year-old woman was referred to our hospital with a chief complaint of a painful mass in the right lower quadrant of her abdomen. She had a history of acute appendicitis, for which she underwent an appendectomy approximately 70 years previously. Imaging studies demonstrated a tumor containing a linear-shaped agent located in the abdominal wall under the surgical scar where the appendectomy was performed. She was then diagnosed with Schloffer’s tumor, for which she underwent surgical resection. However, histopathological examination revealed that the tumor was a fibrous connective tissue mass with a lamellar bone inside.

## INTRODUCTION

Inflammatory granulomas often develop in surgical scars due to the presence of foreign bodies, such as sutures. These granulomas are called Schloffer’s tumor [1]. Endogenous substances are rarely the cause of such tumors. Heterotopic ossification (HO) can develop in incision scars after abdominal surgery. Generally, HO that occurs after abdominal surgery is located in the upper midline incision [2]. Here, we aimed to report a case of HO in an appendectomy scar that formed an inflammatory granuloma following HO infection.

**Figure 1:**
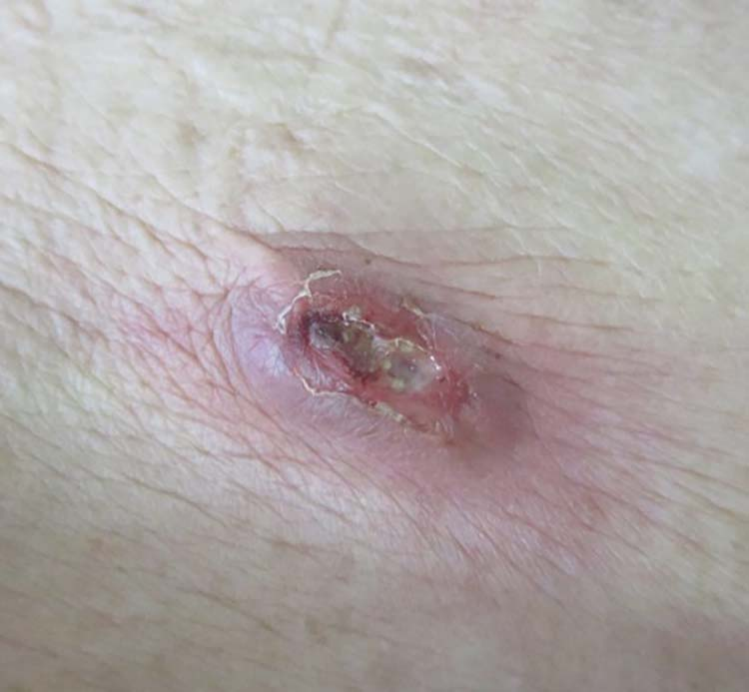
A palpable tumor below the scar of appendectomy was appreciated during the first physical examination, and this eventually caused redness of the skin, which was accompanied by yellowish pus.

## CASE REPORT

**Figure 2:**
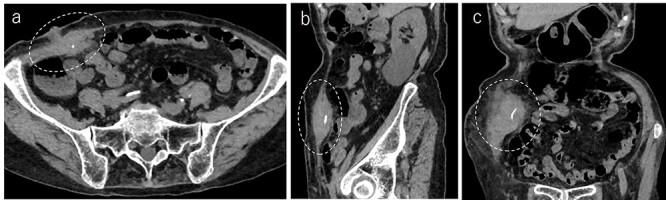
Abdominal CT demonstrated a tumor in the abdominal wall, which extended to the surface of the body (**a**, encircled); the tumor was 60 mm in width and contained a linear shaped agent; sagittal (**b**, encircled) and coronal (**c**, encircled) views.

**Figure 3:**
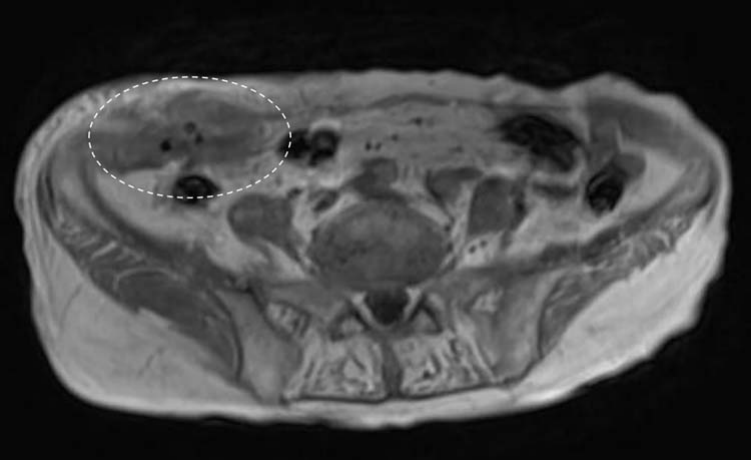
Abdominal MRI showed a low-signal nodule on T1-weighted image (encircled).

A 90-year-old woman was referred to our hospital complaining of a painful mass located in the right lower quadrant of her abdomen. She had a history of acute appendicitis, for which she underwent an appendectomy approximately 70 years prior to the current consultation. She first noticed the pain 6 months prior to consultation. In the interim, the pain gradually worsened. Upon physical examination, a palpable tumor was present below the appendectomy scar. Yellowish pus was removed from the skin over the tumor (Fig. 1). Computed tomography (CT) revealed a tumor in the abdominal wall under the surgical scar of the appendectomy (Fig. 2a). The tumor was 60 mm in width and contained a linear-shaped agent, which was thought to be a foreign body, such as a surgical needle (Fig. 2b and c). Magnetic resonance imaging (MRI) also revealed a low-signal nodule on a T1-weighted image (Fig. 3). Laboratory test results revealed no signs of inflammation. Pus culture revealed no pathogens.

**Figure 4:**
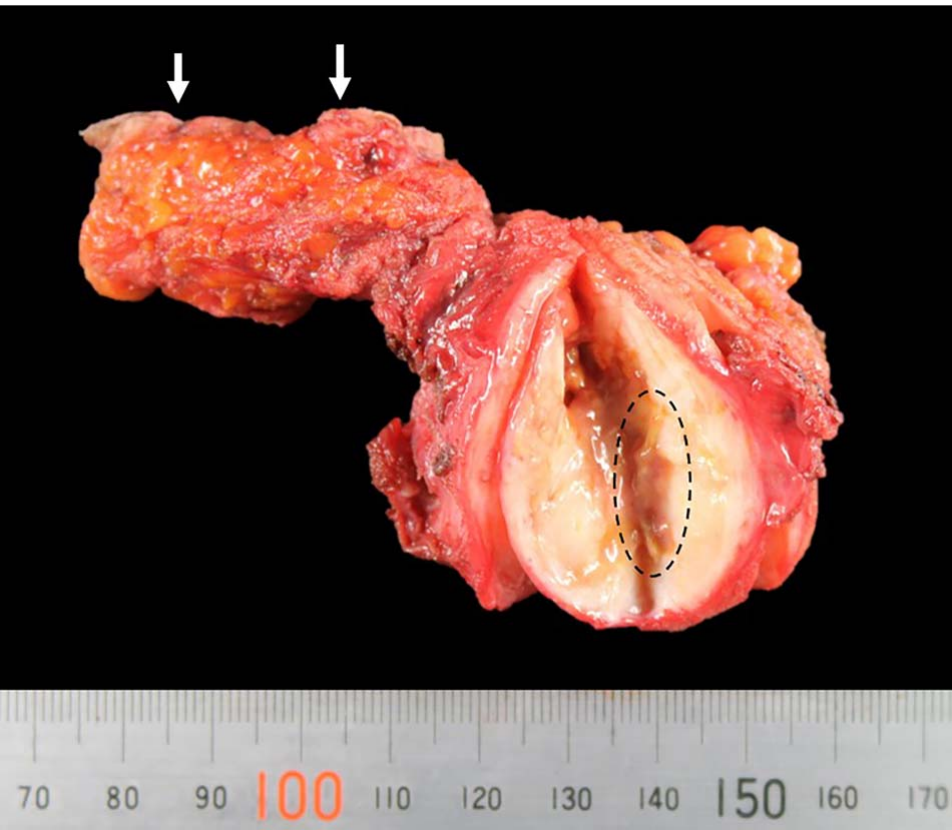
Picture of the resected tumor, fistula and skin(arrow); the tumor contained a stiff and wiry body (encircled).

Under the preoperative diagnosis of foreign body granuloma, also known as Schloffer’s tumors, which contained abscess, the patient underwent surgical resection of the tumor. The tumor was 60 × 40 × 40 mm, slightly hard and capsulized. It also had a stiff and wiry body (Fig. 4).

**Figure 5:**
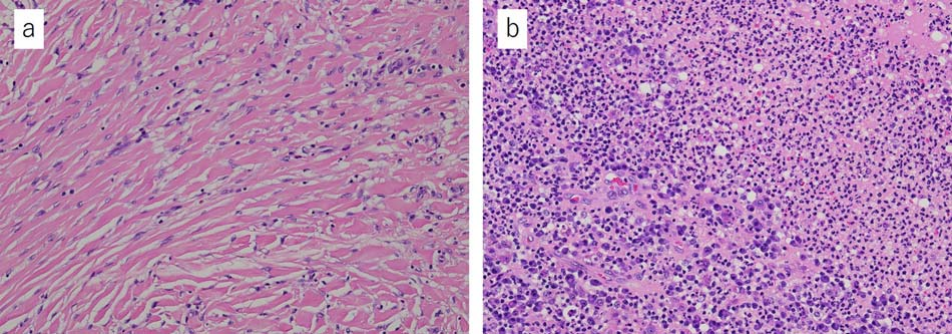
Microscopic findings with hematoxylin and eosin staining of the tumor: (**a**) fibrous connective tissue with diffuse infiltration of neutrophils and histiocytes; (**b**) the tissue contained abscess and bacteria (a ×20, b ×20).

**Figure 6:**
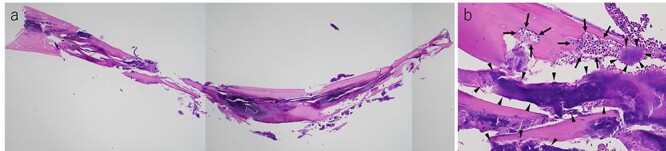
(**a**) Histological finding on hematoxylin–eosin staining of the lamellar bone extracted from the tumor and (**b**) cavity erosion, in which inflammatory cells(arrow) and bacteria (arrow head), were observed (a ×2, b ×20).

Histopathological examination of the tumor revealed fibrous connective tissue with abscesses and bacteria. Diffuse infiltration of inflammatory cells, such as neutrophils and histiocytes, was observed (Fig. 5a and b). The wiry body turned out to be a lamellar bone, in which osteocytes sloughed off (Fig. 6a and b). The lamellar bone had an eroded cavity, in which inflammatory cells and bacteria were present. No artificial matter nor malignancy was observed. The pain was relieved after the surgical resection, and the patient was in good health 4 months after the operation.

## DISCUSSION

Tumors that grow on surgical scars are often diagnosed as Schloffer’s tumor. However, in this case, heterotopic bone tissue, instead of a foreign body, was found in the tumor. Although HO is not common, it can be one of the causes of granuloma development in surgical scars. We speculate that HO developed in the appendectomy scar 70 years after the operation. Thereafter, the infected HO tissue formed a granuloma and an abscess. To the best of our knowledge, there have been no reports of HO accompanied by granuloma generation.

HO is the formation of bone outside the skeletal system, which can occur in all types of soft tissues, such as skin, scars, subcutaneous fat, muscle and mesenterial tissue [3]. Askanazy *et al.* first reported HO in an abdominal scar in 1901 [4]. In a recent study, the postoperative CT scan of 25% of the 152 consecutive patients who underwent abdominal incisions revealed the presence of HO [5]. In Japan, most patients are reported to have developed HO in the upper midline incision, but HO is also found in the transverse or lower incisions in Western countries.

The pathogenesis of HO has not yet been fully elucidated. Nevertheless, several theories have been proposed. Some studies suggested that vertical incisions in the proximity of the xiphoid process or pubic symphysis inoculated periosteal or perichondrial cells. This ‘seeding’ of the surgical wound with activated osteoprogenitor cells could therefore induce formation of heterotopic bone [3]. Other studies suggested a process known as osteogenic induction. Immature pluripotent mesenchymal cells, localized in muscle tissues, differentiate into osteoblasts or chondroblasts and subsequently induce bone formation [6, 7]. Cell differentiation occurs in response to a certain mechanical stimulus or in response to a combination of multiple stimuli, such as chronic inflammation. Inflammation may induce a cascade of biochemical events that ultimately lead to the formation of HO [3].

Recent studies proposed that bone morphogenetic proteins (BMPs), which were involved in the regulation of bone induction, maintenance and repair, played critical roles in osteogenesis and chondrogenesis [8]. Yu *et al.* reported the role of BMPs in heterotopic bone formation, and suggested that inflammation and activation of BMP signaling are necessary for the development of HO [9].

In our case, HO developed in the right lower quadrant of the abdomen.

Since there was no continuation of bone tissue nor periosteum, we assumed that immature pluripotent mesenchymal cells differentiated into bony tissue in the surgical scar. We assumed that surgical invasion, BMPs and inflammation-induced cell differentiation into HO. Although no studies reported a case of an infected HO, bacterial infection of HO tissue seemed to occur through the bloodstream from other sites. Some reports showed that acupuncture causes ectopic infection, thereby resulting in piriformis muscle abscess, trapezius muscle abscess and necrotizing soft-tissue infection [10, 11]. Our patient underwent acupuncture treatment in the past as well, which could be the cause of HO infection.

We initially diagnosed the tumor as Schloffer’s tumor. They are caused by infection of foreign bodies, such as sutures left in the abdominal wall, which then generates granulomas. On the other hand, few patients were diagnosed with Schloffer’s tumor because it existed in the surgical scar, although no foreign body was detected. We concluded that our case was not Schloffer’s tumor because the tumor was derived from an intrinsic lamellar bone.

We performed the surgery for two indications. The first indication was the symptomatic presentation of the patient. The second reason was to arrive at as definitive diagnosis.

## CONCLUSION

We reported a very rare case of infected HO that grew on an appendectomy scar and resulted in an inflammatory granuloma and abscess 70 years after the surgery.

## AUTHOR CONTRIBUTIONS

C.H. and Y.T. substantially contributed to the conception and design of the case report. They also drafted the manuscript. K.M. acquired the pathological data. K.K., M.M., R.T., K.S., T.H., T.D., A.T., K.G., M.M., K.T. and M.H. contributed in drafting the manuscript. T.K. critically revised the manuscript. All authors have read and approved the final manuscript.

## Data Availability

The datasets supporting the conclusions of this article are included within the article.
